# Delayed presentation of vernix caseosa peritonitis

**DOI:** 10.1308/003588412X13373405385296

**Published:** 2012-05

**Authors:** AC Chambers, AV Patil, R Alves, JC Hopkins, J Armstrong, RN Lawrence

**Affiliations:** Great Western Hospitals NHS Foundation Trust,UK

**Keywords:** Vernix caseosa peritonitis, Caesarean section, Laparotomy

## Abstract

**INTRODUCTION:**

Vernix caseosa peritonitis (VCP) is a rare and poorly recognised condition resulting from a sustained foreign body reaction to the vernix caseosa of the baby. This case-based review aims to highlight its importance for any medical team managing patients with peritonitis who have undergone a recent Caesarean section.

**CASE REPORT:**

A 31-year-old woman presented 5 weeks after a Caesarean section with symptoms and signs of peritonitis.

**CONCLUSIONS:**

Laparotomy and peritoneal lavage is the mainstay of treatment for VCP. Knowledge of the condition may stop inadvertent resection of normal intra-abdominal organs. Greater awareness of VCP is required to ensure earlier recognition as patients can recover well following timely operative intervention.

Vernix caseosa is a thick white paste that is present on foetal skin from the third trimester of pregnancy until shortly after birth.^1^ It is unique to our species and consists predominantly of water (80%) combined with proteins and lipids in equal portions (20%). Biologically, vernix caseosa is beneficial to the neonate and infant via production of antimicrobial peptides^2^ as a moisturiser for neonatal skin and providing a waterproof barrier allowing for maturation of the neonatal stratum corneum. It is thought to be the cause of vernix caseosa peritonitis (VCP).^3^

VCP is a rare condition that affects women post-Caesarean section following spillage of amniotic fluid and vernix caseosa into the peritoneal cavity. Vernix caseosa can result in a profound systemic inflammatory response that necessitates maternal laparotomy and may lead to erroneous resection of intra-abdominal organs. Diagnosis is often difficult due to a lack of awareness of the condition and may only be made following histological examination. Confusion regarding diagnosis can result in considerable anxiety for both the patient and surgeon in the ongoing management of the condition.

We present a case report of VCP in a 30-year-old woman 5 weeks after a Caesarean section.

## Case history

A 30-year-old primigravida woman delivered a healthy baby boy by lower segment Caesarean section (LSCS) at 40 weeks after conversion from natural delivery due to failure to progress. She had an unremarkable antenatal period and no significant medical history. Five weeks following the LSCS, she presented with a 48-hour history of generalised abdominal pain, vomiting and diarrhoea, and was admitted with a provisional diagnosis of gastroenteritis under the care of the medical team. Clinical examination revealed a systemic inflammatory response syndrome (SIRS) with a pulse rate of 150bpm, blood pressure of 97/55mmHg and temperature of 39.1ºC. Abdominal examination demonstrated a soft abdomen with generalised tenderness. The LSCS wound looked healthy. The C-reactive protein level was 481mg/l with a neutropaenia of 2.2 × 10^9^/1.

Intravenous fluid and antibiotics were administered. Computed tomography (CT) of the abdomen and pelvis on day 2 demonstrated some free fluid. The patient was transferred to the intensive care unit (ICU) and was prescribed granulocyte colony stimulating factor, which increased her neutrophil count to 41.2 × 10^9^/l. Stool, urine and blood cultures were all negative.

The patient deteriorated further with increased pain, a distended abdomen and signs of peritonitis. Repeat CT demonstrated an increased volume of intra-abdominal free fluid. An urgent laparotomy was performed, which revealed a multiloculated fluid collection in the abdomen and pelvis that was not malodorous. The uterus was intact. There was widespread inflammatory change throughout the peritoneal cavity with large volumes of fibrinous material. Appendicitis was presumed and an appendicectomy and extensive peritoneal lavage performed. Two large drains were sited, in the right paracolic gutter and the pelvis. The patient improved post-operatively with a concomitant decrease in inflammatory markers.

**Figure 1:**
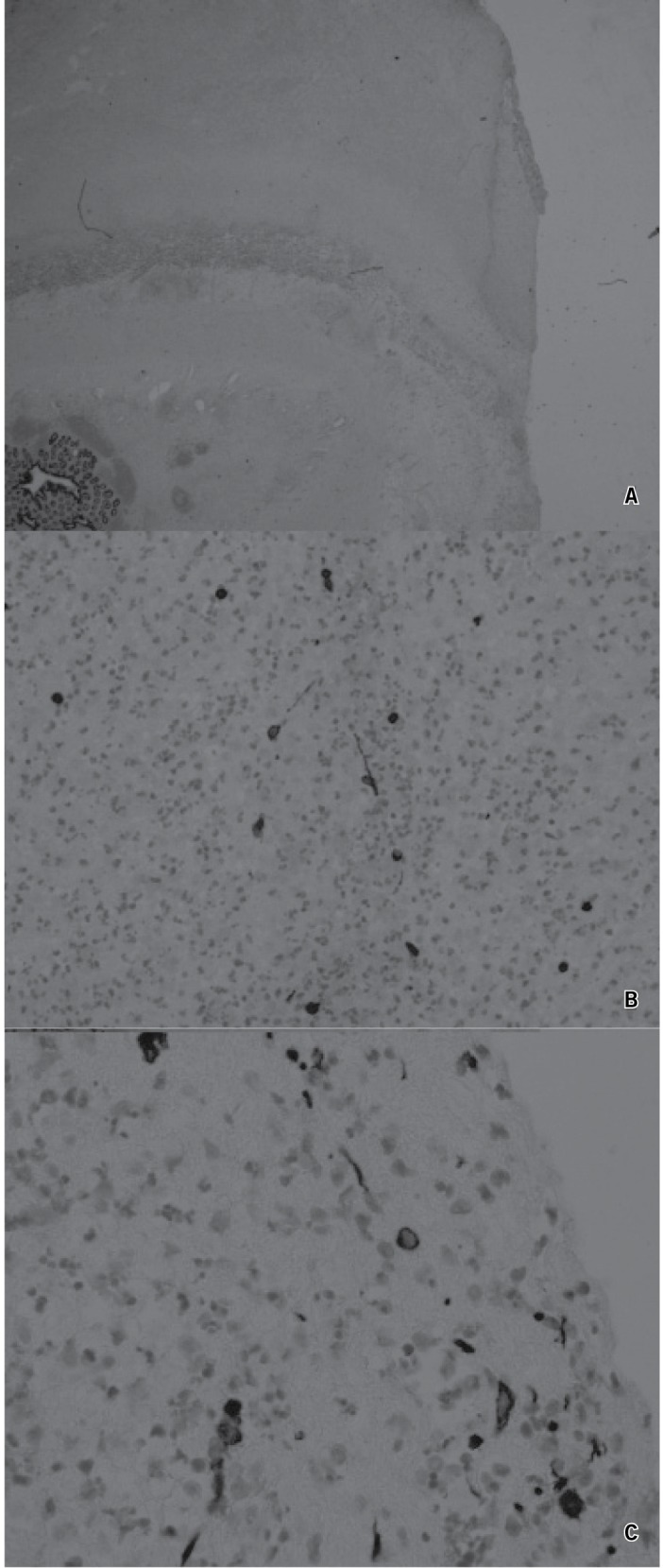
Histology of appendix and intra-abdominal tissue: The pan-cytokeratin stain demonstrates a mesothelial reaction on the serosal surface of the appendix and over the serosal exudates (A; 10x magnification). At higher power one can see both nucleated cell staining with cytokeratin (mesothelial cells) and anucleate linear structures consistent with anucleate squamous cells (B; 200x magnification). Both nucleated mesothelial cells and anucleate cells consistent with squamous cells are seen in the exudates from the peritoneal cavity (C; 400x magnification).

Histology of the appendix revealed acute fibrinous serositis and material taken from the abdominal cavity demonstrated degenerate anucleate squamous cells in keeping with VCP (Fig 1). Further cultures taken from the drain post-operatively revealed no evidence of intra-abdominal infection. The patient was discharged pain free 23 days after surgery.

## Discussion

The literature base for VCP is limited (Table 1) and, to date, only 20 cases of VCP following a Caesarean section have been published.^3–15^ It is unclear whether two of the cases of VCP described by Herz *et al*^5^ had been published previously. The largest series of three patients is from Australia.^16^ Most of the cases, however, were from the US.^17^ VCP has also been seen antenatally following spontaneous rupture of membranes. Caesarean sections performed on two women revealed VCP at the time of delivery, presumably as a result of reflux of vernix caseosa into the peritoneal cavity.^18^

**Table 1:** Case reports of vernix caseosa peritonitis following Caesarean section

Authors	Cases	Caesarean to acute presentation	Operation performed	Resected organs	Histopathology	Microbiology	Outcome
Krumerman and Pouliot, 1976^4^	1	28 days	Laparotomy	Appendix	Appendiceal serositis, giant cells surrounding anucleate squames	Peritoneal material culture -ve	Recovered
Herz *et al*, 1982^5^	2	a) 8 days; b) 14 days	a) Laparotomy + adhesiolysis; b) Laparotomy	a) None; b) Hysterectomy + BSO	a) Granulomatous reaction + squames, lanugo hair, keratin and meconium; b) Tubo-ovarian abscess + squames surrounded by giant cells	a) N/A; b) Exudate cultured *B melaninogenicus*	Recovered
Freedman *et al*, 1982^6^	2	a) 9 days; b) 17 days	a) Laparotomy; b) Laparotomy	a) Appendix; b)Hysterectomy + BSO	a) Foreign body reaction + keratin debris and hair fragments; b) Tubo-ovarian abscess + squames engulfed by foreign body giant cells	N/A	N/A
Boothby *et al*, 1985^7^	1	35 days	Laparotomy	Descending colon	Colon not perforated, giant cells, macrophages with degenerating superficial squamous cells	N/A	Recovered
George *et al*, 1995^8^	2	a) 8 days; b) 10 days	a) Laparotomy; b) Laparotomy	a) Ascending colon; b) Omentum	a) Serositis, granulomatous inflammation with anucleate squamous cells, lanugo hair; b) mixed inflammation with anucleate squamous cells	a) Peritoneal fluid culture -ve; b) Culture -ve	Recovered
Zellers and Balaj, 1996^9^	2	a) 12 days; b) 5 days	a) Laparotomy; b) Laparotomy	a) Appendix; b) Appendix	Both a) and b): Periappendicitis + foreign body reaction with anucleate squamous cells	N/A	Recovered
Nuñez, 1996^10^	1	3 days	Laparotomy	Ascending colon	Not perforated, peritonitis, foreign body granulomas with anucleated squames	N/A	Recovered
Mahmoud *et al*, 1997^11^	1	3 days	Laparotomy	Appendix	Anucleated squamous cells	N/A	Recovered with steroid therapy
Tawfik *et al*, 1998^12^	1	4 days	Laparotomy	None	Mixed inflammatory exudate + squames, hair shafts and meconium pigment	Blood + aspirate cultures -ve	Further abscess drained
Cummings *et al*, 2001^13^	1	6 days	Laparoscopy	Gallbladder	Granulation tissue aggregated about anucleate squames; no acute cholecystitis	Gram -ve	Recovered
Selo-Ojeme *et al*, 2007^3^	1	7 days	Laparotomy	None	N/A	Scanty growth of Gram positive	Recovered
Stuart *et al*, 2009^16^	3	a) 5 days; b) 8 days; c) 4 days	a) Laparotomy; b) Laparotomy; c) Laparoscopic to open + 2nd laparotomy	a) Omental biopsy; b) None; c) Appendix at 2nd operation	a) Serositis, granulomas + fetal squames; b) Chronic granulomas + fetal squames; c) Acute inflammation surrounding fetal squames and keratin debris from 1st operation	a) N/A; b) Urine culture -ve; c) Urine + blood cultures -ve	a) Recurrent vernix caseosa abscesses; b) 7 months’ pain; c) Recurrent obstruction
Wisanto *et al*, 2010^14^	1	24 days	Laparoscopy	None	Anucleated squamous cells	N/A	Recovered
Myers and Fernando, 2011^15^	1	21 days	Fine needle aspiration	None	Mixed inflammatory response with anucleate and mature squamous cells	Culture -ve	Recovered with no operation

-ve = negative; BSO = bilateral salpingo-oophorectomy; N/A = not applicable

Our patient presented to hospital five weeks following her LSCS, which is the longest period currently described in the literature. Diagnosis was made after seven days, multiple radiographic tests, a period on the ICU, and following laparotomy and histological confirmation. This highlights the diagnostic dilemma with VCP.

A study published in 2011 reports that VCP could be diagnosed with image guided biopsy^15^ although this may not be practical in the presence of peritonitis and SIRS. Most women in this cohort of patients, in corroboration with our case, progressed to emergency laparotomy and organ resection for presumed diagnoses of more common causes of peritonitis (appendicitis, bowel/ureteric injury or uterine rupture). Our experience suggests such difficulty in diagnosis can result in considerable psychological stress for the patient.

Following our experience with VCP, we recommend laparotomy and peritoneal lavage as the mainstay of treatment, with intravenous antibiotic cover and critical care support if required. We note that steroid therapy has been used successfully in some cases.^11^ However, in our case we felt it was not appropriate. Prognosis appears to be good with the majority of patients recovering following surgery. Nevertheless, some authors report recurrent vernix caseosa abscess formation necessitating drainage or reoperation.^12,16^

Although the condition is not often described in medical textbooks,^17^ with the increasing number of Caesarean sections performed worldwide, this diagnosis must be considered in post-Caesarean section women presenting with abdominal pain and signs of peritonitis.

## Conclusions

This case highlights the lack of awareness in the medical community of VCP. In spite of its rarity, VCP represents serious post-Caesarean section morbidity and may require urgent surgical management. Diagnosis requires close involvement with the histopathologist. Management should entail rigorous peritoneal lavage and placement of intra-abdominal drains to monitor for early recurrence. It is unknown whether VCP can present later than five weeks post-partum but consideration of VCP as a diagnosis is important in any woman presenting with an acute abdomen following a recent Caesarean section.
